# Update on the pathogenic potential and treatment options for *Blastocystis* sp

**DOI:** 10.1186/1757-4749-6-17

**Published:** 2014-05-28

**Authors:** Tamalee Roberts, Damien Stark, John Harkness, John Ellis

**Affiliations:** 1 Department of Microbiology, St. Vincent’s Hospital, Victoria St, Darlinghurst 2010, NSW, Australia; 2 School of Medical and Molecular Biosciences, University of Technology, Sydney, Ultimo, NSW 2007, Australia

**Keywords:** Blastocystis, Pathogenicity, Treatment failure, Subtypes, Parasitology

## Abstract

Although *Blastocystis* is one of the most common enteric parasites, there is still much controversy surrounding the pathogenicity and potential treatment options for this parasite. In this review we look at the evidence supporting *Blastocystis* as an intestinal pathogen as shown by numerous case studies and several *in vivo* studies and the evidence against. We describe the chronic nature of some infections and show the role of *Blastocystis* in immunocompromised patients and the relationship between irritable bowel syndrome and *Blastocystis* infection. There have been several studies that have suggested that pathogenicity may be subtype related. Metronidazole is the most widely accepted treatment for *Blastocystis* but several cases of treatment failure and resistance have been described. Other treatment options which have been suggested include paromomycin and trimethroprim- sulfamethoxazole.

## Introduction

**Figure 1:**
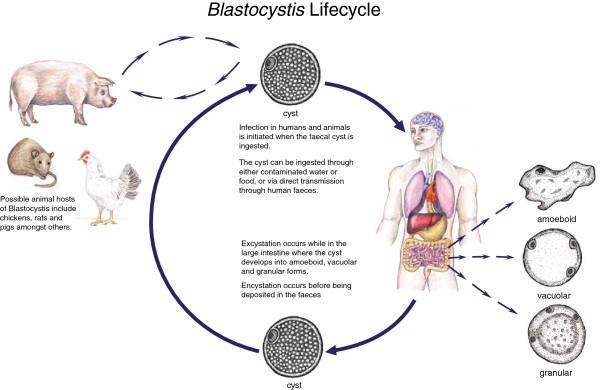
**Lifecycle of *Blastocystis *** **sp.**

*Blastocystis* is one of the most common intestinal protists of humans. *Blastocystis* was first described 100 years ago but surprisingly little is still known about the pathogenicity, genetic diversity, host range and treatment. First classified as yeast, *Blastocystis* was then subsequently classified as a protist and has now been placed within the Stramenopiles [1-5]. *Blastocystis* has a world-wide distribution with higher numbers being found in developing countries probably due to poor sanitation [6]. *Blastocystis* has been found in a wide range of animals including mammals, birds and amphibians. Up to 17 subtypes have been described with subtype (ST) 1–9 being found in humans [7]. ST3 is the predominant ST found in most human epidemiological studies [8-10]. Due to the lack of knowledge about this parasite, there is still controversy about whether to treat infections as they may just be opportunistic colonisation. There has been conflicting results about the efficacy of treatments and this is an area where much more research is needed. *Blastocystis* is transmitted by the faecal oral- route by human- human or animal- human transmission. There have been several studies that have shown possible transmission by contaminated water and it has been stated that the poor provision of basic amenities plays an important role in transmission [11-13]. A recent study showed that 100% of people from low socio-economic villages in Senegal were infected with *Blastocystis* sp. suggesting that transmission was increased due to poor hygiene sanitation, close contact with domestic animals and livestock, and water supply directly from well and river [10]. There are several methods for the detection of *Blastocystis*. Microscopy of a permanent stain is the gold standard for the diagnosis of *Blastocystis* in most clinical laboratories. Microscopy was shown to have the lowest sensitivity for the detection of *Blastocystis* (48%) with PCR being the most sensitive technique used (94%) [14]. Figure 1 describes a current view of the lifecycle of *Blastocystis*.

### Pathogenicity

There is still much debate about the pathogenicity of *Blastocystis* in humans. Though many authors have given credit to it as a pathogen [15-18], there are still many that doubt the role of *Blastocystis* in human disease [19,20]. The most common symptoms associated with *Blastocystis* infection include diarrhoea, abdominal pain and vomiting. There are many reports of single patients that show there was no other cause of sickness identified in patients, with *Blastocystis* being the only infection detected.

There have been several case reports suggesting that *Blastocystis* is related to urticaria [4]. The amoeboid forms of *Blastocystis* ST3 were found in a case of acute urticaria and the authors suggested that cutaneous symptoms may be caused by disruptions to the immune homeostasis as the host produces an inflammatory response against the amoeboid forms [21]. Another case showed the presence of *Blastocystis* ST2 in a severe case of gastrointestinal symptoms and chronic urticaria in the absence of any other infectious agent. Symptoms persisted after initial antibiotic therapy but were finally eradicated after combined metronidazole and paromomycin treatment [22]. A recent retrospective study reported 8/80 (11%) *Blastocystis* infected patients to have skin manifestations as well as gastrointestinal symptoms [23]. Unfortunately this study relied solely on microscopy, so no information on ST related to cutaneous lesions can be gathered; however all of these studies do show the potential for *Blastocystis* to cause cutaneous symptoms. Case reports are summarised in Table 1.

**Table 1:** **Case reports of *Blastocystis *** **infection**

**Patient**	**Clinical history**	**Diagnosis**	**Treatment**	**Outcome**	**Study**
One 11 y.o male and one 12 y.o male	Both presented with right lower quadrant tenderness, anorexia, abdominal pain, nausea, vomiting	Initially diagnosed with appendicitis. Stool examination showed *Blastocystis* and patients were then diagnosed with *Blastocystis* infection	Metronidazole and co-trimoxazole	Complete recovery	[15]
24 y.o female	Nine week history of urticaria, hives, chronic diarrhoea, IBS	Initially diagnosed with cellulitis. Treated with non-steroidal cream with no recovery of symptoms. Presented with hives and diagnosed as urticaria. Extensive investigation showed 4 + *Blastocystis* in her stool.	Metronidazole	All urticarial and IBS symptoms cleared	[24]
45 y.o female	Four month history of erythematous and pruriginous lesions on trunk and limbs, mild gastoenteric complaints	Diagnosed with urticaria. Extensive investigation showed the stool postive for *Blastoycstis*.	Paromomycin and metronidazole	All urticarial and gastrointestinal symproms cleared	[25]
32 y.o female	Four year history of allegic rhinitis and chronic urticaria, swelling in pressure sites	Diagnosed with delayed pressure urticaria. Treated with systemic corticosteroids with only partial clearance of symptoms. Stool examination positive for *Blastocystis*	Metronidazole	All urticarial symptoms cleared	[26]
60 y.o female	Four year history of anaphylactoid reactions, severe asthma and generalised urticaria	Diagnosed with chronic urticaria. Extensive investigation identified *Blastocystis* in the stool	Paromomycin	All urticaria symptoms cleared	[27]
74 y.o male	Diarrhoea, abdominal pain, nausea, fatigue and fever	Hospitalised. Stool were positive for *Blastocystis*	Metronidazole	Clearence of symptoms after 10days	[18]
29 y.o female	Six month history of morning stiffness, pain and swelling of joints, elbows, ankles, knees, diarrhoea, abdominal pain and vomiting	Treated for presumed infectious arthritis of the knee. No improvement. Microscopy of the synovial fluid and stools both showed the presence of *Blastocystis*.	Metronidazole	After two weeks knee inflammation subsided and all abdominal pain and diarrhoea were cleared	[28]
24 y.o male	Six week history of diffuse abdominal pain and diarrhoea	Stool examination was positive for *Blastocystis* and *Endolimax nana*.	Metronidazole	Complete resolution of symptoms 10days later	[29]
19 y.o male	Three week history of hives, abdominal pain for 2.5months	Diagnosed with acute urticaria. Routine testing showed the presence of *Blastocystis* in the stool ST3	Metronidazole	10days after treatment both urticaria and abdominal discomfort were cleared	[21]
20 y.o male	Urticaria and flatulence	Treated with antihistamines with no success. Further investigation showed *Blastocystis* ST2 in the stool. Initially treated with metronidazole but treatment failure appears to have occured. Then treated with co-trimoxazole with no success and finally treated with combination metronidazole and paramomycin	Metronidazole then co-trimoxazole followed by paromomycin	All symptoms cleared 10 days later	[22]
40 y.o female	Hospitalised due to severe diarrhoea and fever	*Blastocystis* ST8 infection diagnosed from stool cultures. Treated with metronidazole. Symptoms persisted and the patient also noted bloating, flatulence and abdominal pain. Further treated with co-trimoxazole	Metronidazole then co-trimoxazole	All symptoms cleared	[30]

It was recently suggested that gastrointestinal symptoms related to *Blastocystis* might be ST related but results remain inconclusive [8,31-33]. It was suggested that ST1 may be related to pathogenicity with a higher subtype-symptom relationship being noted [34].

There have been conflicting reports on the pathogenicity of ST2 with some studies showing high symptom- infection rates [22,33] whereas others have seen no link [35,36]. A study in Colombia showed that 100% of patients with diarrhoea had ST2 where asymptomatic people all had ST1 [37]. There have been two previous studies that have suggested ST4 to be a pathogenic strain due to the high incidence of this ST in patients with severe diarrhoea [38,39]. It was also suggested that ST8 could be a pathogenic strain. ST8 is a rare subtype found in humans and in two studies has been related to severe symptoms [8,30]. Even though ST3 is the most common ST found in humans, there is a low association between ST and symptoms shown by patients [8]. An animal study in rats showed that ST1 was statistically related to pathogenicity and that there may be pathogenic and non- pathogenic strains within ST3 and ST4 [40]. These studies highlight the need for more research in to the relationship between ST and symptoms.

The whole genome for ST7 has been described [41]. This genome has shed light on some important processes identifying genes coding for proteins that are responsible for host protease inhibition. Proteins like these may modulate the host protease activity thereby disturbing the intestinal homeostasis [42]. Further information will be gathered as more genomes are described that can help determine the role of genes in the potential pathogenicity of *Blastocystis*.

The study of host immunity to *Blastocystis* is under represented in the literature and offers many opportunities for future study. For example, the study of physiological or genetic factors of the host that may affect the outcome of *Blastocystis* infection as a possible pathogenic organism.

### Immune response and animal studies

Several lines of evidence shed light on the possible mechanisms of pathogenesis. *Blastocystis* express cysteine proteases which have been shown to be sensitive to the inhibitors iodoacetamide and E-64 in azoceasein assays [43]. Cysteine proteases play important functional roles in invasion of host cells, immune evasion, pathogenesis, virulence and cell cycle regulation. It was shown that proteases from *Blastocystis* isolates can degrade human secretory immunoglobulin A [44] and that *Blastocystis* WR1 ST4 induces contact-independent apoptosis, F-actin rearrangement and barrier function disruption in IEC-6 cells [45]. There was shown to be extensive variation in morphology and protease activity between the two different STs, 4 and 7, of *Blastocystis*[46] with the avian (ST7) isolates having almost twice as much cysteine protease activity compared to the rodent (ST4) isolates. These two STs were also shown to cleave secretory IgA with cysteine and aspartic protease activities respectively. These results suggest the possibility of *Blastocystis* proteases as virulence factors and that they contribute to parasite survival *in vivo* by degrading neutralising mucosal antibodies. Another study was able to identify two cysteine proteases (a cathepsin B and a legumain) secreted by ST7 which could be helpful in the development of virulent and diagnostic markers as well as targets for chemotherapy [47]. One study suggested that 32 kDa proteases of ST3 could be virulence factors responsible for protein degradation [48] while another study suggested that the 29 kDa *Blastocystis* antigen could be used as a marker for pathogenicity and differentiate between symptomatic and asymptomatic infections [49]. Higher levels of IgA in symptomatic individuals with *Blastocystis* compared to healthy asymptomatic carriers has also been described [50]. A recent study on the effect of *Blastocystis* on the expression of interferon gamma and proinflammatory cytokines in the cecal mucosa of rats showed a significantly upregulated amount of gene transcription of type 1 and proinflammatory cytokines IFN-γ, IL-12 and TNF-α. This suggests that *Blastocystis* infection in rats stimulates specific local host responses including T cells, monocytes/macrophages and/or natural killer cells when exposed to antigens [51]. Several mice studies have highlighted the effect of *Blastocystis* on infected mice with weight loss and diarrhoea occurring when mice were inoculated with high doses of *Blastocystis*[52-54]. Another study showed that *Blastocystis* can invade the lamina propria, submucosa and muscle layers [55] while another study found elevated levels of hyaluronidase in rat urine infected with *Blastocystis* which suggests invasion of the colonic epithelium with *Blastocystis* but further investigation in humans is necessary to confirm this [56]. One study highlighted the use of laboratory rats as a good animal model for *Blastocystis* infection. They showed how rats infected with ST1 showed histopathological changes at all the different doses given and suggested that ST1 infection has pathogenic potential with individual variation [57]. These studies show how animals can be used as a good model for pathogenicity but it is important to take in to consideration that mice are not naturally infected with *Blastocystis* unlike rats which are commonly found to harbour *Blastocystis*.

### Blastocystis infection in immunodeficient patients

Infections of the gastrointestinal tract play a fundamental role in the morbidity and mortality of acquired immunodeficiency syndrome (AIDS) and human immunodeficiency virus (HIV) patients. A much higher rate of gastrointestinal tract infections has been described since the first cases of HIV and AIDS were reported including diarrhoea associated with parasitosis [58,59]. Diarrhoea is one of the clinical manifestations in HIV infection and usually tends to be chronic. Parasite induced diarrhoea is prominent in AIDS patients and there is varying infection rates due to geographical location with a high incidence in developing countries (e.g. up to 95% of infected people in Africa and only up to 50% in developed countries). Suppressed immunological responses at the mucosal level that hinder the intestinal non- specific defence mechanisms in the gastrointestinal tract play a major role in AIDS pathogenesis.

There have been several studies into the prevalence of intestinal parasites in HIV and AIDS infected people with varying results and in particular the incidence of *Blastocystis* in these study populations. A study in Brazil found 40% of patients in an HIV positive population to be infected with at least one enteropathogen and some with two or more present [59]. In this study though, only one patient was infected with *Blastocystis* which suggests that this protozoan may not be an opportunistic parasite in HIV infected people. Another study in northern India found only two patients (7.7%) of the study population to be infected with *Blastocystis* with 19 of the 26 people studied having parasitic infections [60]. Though this is not a high incidence of the parasite in the population, it was shown that in these two patients there were 10 or more organisms seen per field of view and the presence of no other pathogens suggested that *Blastocystis* was the cause of diarrhoea in these patients. This is in comparison to studies done in Africa which showed *Blastocystis* infection to be at a higher rate in HIV positive patients compared to a control group. A study in Senegal found *Blastocystis* in only HIV infected patients with all but one suffering from diarrhoea and with no other pathogens found in the samples. This study suggested that *Blastocystis* should be considered an opportunistic parasite [61]. Another African study in an Ethiopian teaching hospital found there to be an incidence of 14.1% of *Blastocystis* infection in HIV/AIDS patients. There were no statistically significant differences in the prevalence of parasites amongst cases and controls except that of *Blastocystis* which was significantly higher in HIV/AIDS patients. They concluded that *Blastocystis* was a possible pathogenic agent in immunocompromised patients [62]. A more recent study of intestinal parasites in HIV/AIDS patients in Ethiopia showed that *Blastocystis* was the third most common parasite identified at 10.6% of the study population of 248 patients [63]. There were no *Blastocystis* infections seen in the HIV negative group. Diarrhoea was a clinical finding in 80.9% of the parasite positive patients. Another study in Ethiopia showed that the presence of intestinal parasite infections were significantly higher among HIV positive people not on Antiretroviral Treatment (ART) compared to those on ART [64]. *Blastocystis* was the second most common parasite identified in the non ART group at 12.8% positive and there was a significant association between *Blastocystis* infection and symptoms of diarrhoea. A study in Iran showed that the occurrence of parasites in HIV positive patients was not as high as seen in African countries with an infection rate of only 18.4%. Of the parasites seen in this study though, *Blastocystis* was the second most prevalent at 4.4% with most of these cases being seen in diarrhoea positive patients [65]. In Indonesia a total of 318 HIV positive patients were investigated for parasites and *Blastocystis* was identified as the most common parasite occurring in 73.6% of the patients [66]. *Blastocystis* was found to be present in all CD4^+^ groups with either high or low counts. A study in China identified *Blastocystis* as the most common enteric parasite in both the HIV positive and HIV negative groups but it was observed that there was actually a higher percentage in the HIV negative group [67]. This study also observed that co-infection with *Blastocystis* and HIV created lower CD4 levels and higher IL-2 levels compared to the other co-infections with parasites. A recent study on the STs found in HIV/AIDS patients identified 19.8% of patients positive for *Blastocystis* with ST3 being the most common subtype with 55% of isolates followed by ST4 with 25%, ST1 with 15% and ST2 with 5% [68]. The majority of isolates belonging to ST3 is consistent with results from most molecular epidemiological studies conducted around the world.

Most of these studies show that *Blastocystis* is not higher in the HIV/AIDS population than what was previously found in normal populations with *Blastocystis* incidence ranging from 6-70% in developing counties. There are also some problems in relation to accuracy of these results with most studies relying on techniques such as the less sensitive microscopy and culture. Although these studies give varying results in respect to *Blastocystis* infection in HIV/AIDS patients, this parasite should still be considered as a cause of diarrhoea in these cases and shows the significance of parasite infection in immunosuppressed patients.

A study on cancer patients and *Blastocystis* infection showed that *Blastocystis* was acquired after the commencement of chemotherapy treatment. This study raises the possibility of opportunistic infections of *Blastocystis* in immunocompromised people [69]. Another study showed that 7.7% of cancer patients were infected with *Blastocystis* with a slightly higher rate of detection found in the pre-treatment group (9.7%) as opposed to the post-treatment group (6.7%) [68]. Another study in France compared the occurrence of *Blastocystis* in immunocompromised patients with haematological malignancies (HM) and a non immunocompromised control group. The study showed that there was not a high level of difference between the two groups with prevalence values of 16% for the HM group and 13% for the control group but there was a difference in STs found within the groups. ST4 was the most common ST found in both the HM and control group (66.7% and 58.3% respectively) followed by ST3 (20%), ST6 (6.7%) and ST7 (6.7%) in the HM group. In the control group the second highest was ST7 (16.7%) followed by equal number of ST1, ST2 and ST3 (8.3%) [70]. These studies show how *Blastocystis* can easily be an opportunistic infection.

### Irritable bowel syndrome and the role of *Blastocystis*

There have been several hypotheses and increasing studies in the last few years relating the incidence of *Blastocystis* infections with the prevalence of irritable bowel syndrome (IBS) in patients. Due to *Blastocystis* causing symptoms similar to those attributed to IBS such as diarrhoea, abdominal pains and cramps and nausea it is easily seen why an association with this parasite and IBS patients could be made. It is also possible that the change in the environment in the intestine caused by IBS may allow for the conditions favoured by *Blastocystis* for growth. It has been proposed that a possible mechanism for the IBS- like symptoms might be the low-grade inflammation through persistent antigenic exposure in a chronic *Blastocystis* infection [71]. It has also been suggested that polymorphisms in genes encoding inflammatory cytokines might have a role in the pathophysiology of IBS. A recent study has suggested that there is a role in the etiology of IBS from the association between IL-8 and IL-10 gene polymorphisms in IBS- *Blastocystis* carriers [72]. One study showed a possible link between *Blastocystis* and IBS (with 95 IBS patients and 55 control cases) where there was an infection rate of 46% in IBS patients and only 7% in the control group was shown [73]. There have been several other studies which have shown the high number of *Blastocystis* positive individuals in the IBS group compared to the control group with rates of 71%, 76% and 49% with less than 20% in the control groups [74-76].

A recent study performed in Mexico on IBS patients showed an association between *Blastocystis* and pathogenicity with 31% of IBS patients found to harbour *Blastocystis*[77]. This study showed a high number of ST1 and ST3 infections within this population which are also common in most non- IBS populations. This study therefore does not show an association between subtype and IBS infection. A different study on the STs associated with IBS showed a much higher incidence of ST1 in the IBS group compared to the control group but with an equal number of ST3 from both groups [74]. Another study from Egypt highlights the prevalence of ST1, ST3 and ST4 in IBS patients with ST1 only being detected in the IBS group and not the control group and also showed that ST1 was statistically more relevant to pathogenicity than the other STs [78]. In Colombia, 100% of IBS patients with *Blastocystis* were identified as harbouring ST3 [37]. The differences from these studies highlights that more research needs to be done on IBS and *Blastocystis* STs associated with disease but does suggest that there may be a role of *Blastocystis* in IBS.

### Treatment

Due to the controversy surrounding the potential pathogenicity of *Blastocystis* and the self-limiting nature of symptoms, the treatment of this disease is equivocal. Metronidazole is the most frequently prescribed antibiotic for infections [24,79,80]. Various drug treatments using metronidazole have been prescribed ranging from 250–750 mg three times a day for 10 days [81,82] or used in combination with other drugs including paromomycin [25] or trimethroprim- sulfamethoxazole (TMP-SMX) [15]. There have been reports of resistance to metronidazole [83,84] and the cyst form has been shown to have resistance up to 5 mg/ml [85]. Nitazoxanide, a broad-spectrum 5-nitrothiazole antiparasitic agent has also been reported to be effective in treatment [86,87]. Other studies have shown the efficacy of emetine, furazolidone, TMP-SMX, iodochlorhydroxyquin and pentamidine [80,88]. One study also showed the potential benefits of *Saccharomyces boulardii* treatment on *Blastocystis* infected children in Turkey [89]. A case study in Australia of 18 patients showed that clearance of *Blastocystis* and symptoms did not occur after treatment with either metronidazole, iodoquinol or triple combination therapy consisting of nitazoxanide, furazolidone and secnidazole showing the lack of efficacy of several commonly used antimicrobials for the treatment of *Blastocystis*[90]. Table 2 summarises the efficacy of antibiotics from previous studies. This table shows the large variation and contradictory results from the different studies with the same antibiotic dose having different efficacies in different studies.

**Table 2:** **Summary of treatments and efficacy for *Blastocystis *** **infection**

**Treatment (Dose)**	**Efficacy**	**Reference**
Iodoquinole (650 mg t.i.d)	0%	[91]
Emetine (100 μg/ml)	50%	[92]
Metronidazole (2000 mg s.i.d)	0%	[93]
Metronidazole (1500 mg s.i.d)	100%	[26]
Metronidazole (750 mg t.i.d)	100%	[24]
Metronidazole (750 mg t.i.d)	100%	[21]
Metronidazole (500 mg t.i.d)	100%	[79]
Metronidazole (250- 750 mg t.i.d)	33%	[80]
Metronidazole (750 mg t.i.d)	100%	[81]
Metronidazole (1500 mg s.i.d)	80%	[94]
Metronidazole (800 mg t.i.d)	0%	[30]
Metronidazole (30 mg/kg twice daily)	67%	[89]
Nitazoxanide (500 mg t.i.d)	100%	[86]
Nitazoxanide (100-200 mg b.i.d for children <12 yr, 500 mg b.i.d for >11 yr)	86%	[88]
Nitazoxanide (500 mg t.i.d)	100%	[95]
Ornidazole (500 mg t.i.d)	50%	[96]
Paromomycin (25 mg/kg t.i.d)	100%	[93]
Paromomycin (500 mg t.i.d)	100%	[97]
Paromomycin (25 mg/kg t.i.d)	100%	[82]
Paromomycin (1000 mg b.i.d) & MZ (750 mg t.i.d)	100%	[25]
*Saccharomyces boulardii* (250 mg b.i.d)	78%	[89]
Trimethroprim-SMX	22%	[80]
Trimethroprim-SMX (6 mg/kg TMP, 30 mg/kg SMX s.i.d)	95%	[98]
Trimethropim- SMX (320 mg TMP, 1600 mg SMX s.i.d)	93%	[98]
Trimethroprim- SMX (80 mg TMP, 400 mg SMX t.i.d)	100%	[30]
Triple therapy (nitazoxanide, furazolidone and secnidazole)	0%	[90]

It has also been proposed that the different STs of *Blastocystis* have varying susceptibility to antimicrobial drugs [99]. There have been four *in vitro* studies looking at susceptibility patterns of *Blastocystis*. Although these studies had a small number of study isolates, it was apparent that different STs show different susceptibility patterns and that metronidazole is not the most effective treatment for *Blastocystis* infection [92,100-102].

Due to the uncertainty of whether this parasite is a pathogen or not does make it difficult for physicians to decide whether to treat the infection. There are several online resources including The Blastocystis Research Foundation (http://www.bhomcenter.org) which is helpful for both physicians and patients with information about symptoms and treatments and also presents some of the implications of infection in relation to transmission within families and households.

Treatment should be considered if there are chronic symptoms of diarrhoea and abdominal pain in the absence of other pathogens identified from the stool sample. Metronidazole should not necessarily be considered first line treatment due to the large number of cases of treatment failure and other antimicrobials such as trimethroprim- sulfamethoxazole. There may be a correlation between ST and sensitivity to drugs which is yet to be addressed in studies.

## Conclusions

It is clear from all these studies that there is still much work to be done in the areas of pathogenicity, treatment and control. As more information from the genome is gathered there will be more opportunity to identify possible genes that transcribe for pathogenicity and treatment resistance. A simple antimicrobial susceptibility test for *Blastocystis* could help to assure the correct drug is administered and not allow for resistance to develop. Diagnosis should be made by the use of PCR and treatment should be considered when no other infectious agent can be identified.

## Competing interests

The authors declare that they have no competing interests.

## Authors’ contributions

TR was involved in all aspects of the manuscript including acquisition and interpretation of data, drafting of manuscript and making final revisions. DS, JH and JE were all involved in revisions of the manuscript and reviewing of data. All authors have given approval of this final version.
